# Glyteer, Soybean Tar, Impairs IL-4/Stat6 Signaling in Murine Bone Marrow-Derived Dendritic Cells: The Basis of Its Therapeutic Effect on Atopic Dermatitis

**DOI:** 10.3390/ijms19041169

**Published:** 2018-04-12

**Authors:** Masaki Takemura, Takeshi Nakahara, Akiko Hashimoto-Hachiya, Masutaka Furue, Gaku Tsuji

**Affiliations:** 1 Department of Dermatology, Graduate School of Medical Sciences, Kyushu University, Maidashi 3-1-1, Higashiku, Fukuoka 812-8582, Japan; take0917@dermatol.med.kyushu-u.ac.jp (M.T.); nakahara@dermatol.med.kyushu-u.ac.jp (T.N.); ahachi@dermatol.med.kyushu-u.ac.jp (A.H.-H.); furue@dermatol.med.kyushu-u.ac.jp (M.F.); 2 Division of Skin Surface Sensing, Graduate School of Medical Sciences, Kyushu University, Maidashi 3-1-1, Higashiku, Fukuoka 812-8582, Japan; 3 Research and Clinical Center for Yusho and Dioxin, Kyushu University, Maidashi 3-1-1, Higashiku, Fukuoka 812-8582, Japan

**Keywords:** aryl hydrocarbon receptor, Ccl17, Ccl22, dendritic cell, atopic dermatitis

## Abstract

Atopic dermatitis (AD) is a common inflammatory skin disease. Recent studies have revealed the involvement of T helper (Th)2 cytokines including Interleukin 4 (IL-4) in the pathogenesis of AD. Since epidermal Langerhans cells (LCs) and dermal myeloid dendritic cells (DCs) produce CCL17 and CCL22 that chemoattract Th2 cells, interfering with CCL17 and CCL22 production from LCs and dermal myeloid DCs may be beneficial in the treatment of AD. To investigate this, we stimulated murine bone marrow-derived DCs (BMDCs) with IL-4. IL-4 stimulation produced Ccl17 and Ccl22, which was attenuated by soybean tar Glyteer, a known aryl hydrocarbon receptor (Ahr) activator. Notably, Glyteer treatment blocked the nuclear translocation of Stat6 induced by IL-4 stimulation, suggesting that this treatment impairs the IL-4/Stat6 signaling pathway in BMDCs. Unexpectedly, Glyteer treatment did not potently upregulate the expression of Cyp1a1, a specific Ahr-responsive gene, suggesting that its inhibitory machinery for Ccl17 and Ccl22 expression is likely to operate in an Ahr-independent manner. These findings indicate that Glyteer may exhibit therapeutic potential for AD by downregulating the CCL17 and CCL22 production from DCs in a Th2-deviated microenvironment.

## 1. Introduction

Atopic dermatitis (AD) is a common inflammatory skin disease. Its pathogenesis is thought to be derived from cells of the adaptive immune system, mainly T helper (Th)2 lymphocytes [1]. Th2 cytokines such as Interleukin (IL)-4, IL-5 and IL-13 contribute to the development of AD in a STAT6-dependent manner [2]. Dendritic cells (DCs) also play an important role in the pathogenesis of AD, since they can mediate T-cell polarization and produce proinflammatory cytokines and chemokines [3]. DCs serve as sentinels of the immune system and represent a heterogeneous family of myeloid cells. In the skin, the main cell types of myeloid DCs are Langerhans cells (LCs) in the epidermis and dermal myeloid DCs in the dermis [4]. Several studies have revealed that LCs and dermal myeloid DCs produce prominent CCL17 and CCL22 in the skin of AD patients [4,5,6]. CCL17 and CCL22 are considered to be AD-related chemokines produced by skin myeloid DCs [5,6], which chemoattract Th2 cells and maintain the Th2 immune response [5]. Therefore, interfering with CCL17 and CCL22 production from LCs and dermal myeloid DCs may be beneficial in the treatment of AD.

The current standard therapies for AD using immune modulating agents such as glucocorticosteroids, tacrolimus and cyclosporin A are very useful to control the disease activity [7]; however, in some AD patients, such treatment is difficult due to adverse events, suggesting the need for a novel agent that can down-modulate the immune system in AD. Recent studies including ours have revealed the significance of aryl hydrocarbon receptor (Ahr) activators such as coal tar and soybean tar (Glyteer) in the treatment of AD [8,9,10]. Although coal tar and Glyteer are occasionally not tolerated by patients because of the smell and irritation, they improve AD skin conditions [8,9,10]. In addition, treatment of AD with ultraviolet (UV) irradiation is well established and represents a standard second-line treatment [11]. UV irradiation generates 6-formylindolo[3.2-b]carbazole (FICZ) from intracellular tryptophan, which is is a potent endogenous Ahr activator [12]. Therefore, the activation of Ahr by FICZ is an integral part of therapeutic responses by UV irradiation for AD. Indeed, our previous studies have shown that Glyteer and FICZ restore skin barrier functions in AD in an Ahr-dependent manner [9,10,12]. Furthermore, coal tar and Glyteer have been reported to be effective in the treatment of psoriasis [13,14]. It has been shown that activation of AHR exhibits an anti-inflammatory effect via nuclear factor (erythroid-derived 2)-like 2-mediated machinery in human keratinocytes [15].

Much attention has recently been paid to the diverse effects of Ahr signaling in the molecular biology of keratinocytes because Ahr is highly expressed in keratinocytes in the skin [16,17,18,19,20]. Glyteer has previously been revealed to exhibit anti-inflammatory effects and to upregulate filaggrin expression by acting on the AHR in human keratinocytes [10]. It has also been reported that DCs express Ahr and that the activation of Ahr affects the maturation and functions of DCs [21,22]. However, the effects of Glyteer on Ccl17 and Ccl22 expression in DCs have never been reported. We found that Glyteer inhibited the IL-4-induced upregulation of Ccl17 and Ccl22 by blocking IL-4/Stat6 signaling.

## 2. Results

### 2.1. IL-4 Stimulation Induced Ccl17 and Ccl22 Production in BMDCs

**Figure 1:**
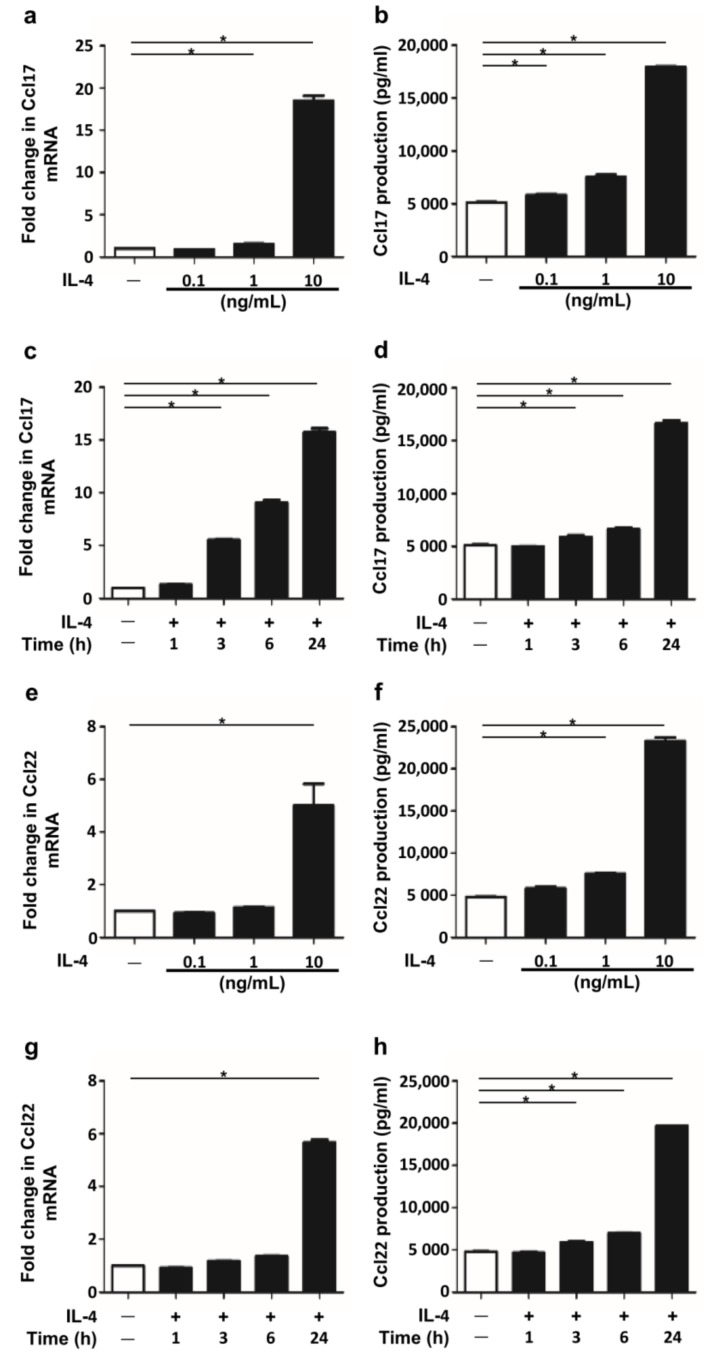
IL-4 stimulation induced Ccl17 and Ccl22 production in bone marrow-derived dendritic cells (BMDCs). (**a**–**d**) Data are expressed as mean ± standard error of the mean (S.E.M.); *n* = 3 for each group; * *p* < 0.05. Expression of Ccl17 (**a**) and Ccl22 (**e**) in BMDCs stimulated with IL-4 (0.1, 1 and 10 ng/mL) for 24 h and production of Ccl17 (**b**) and Ccl22 (**f**) in the culture supernatant were measured. Expression of Ccl17 (**c**) and Ccl22 (**g**) in BMDCs stimulated with IL-4 (10 ng/mL) for 1, 3, 6 and 24 h and production of Ccl17 (**d**) and Ccl22 (**h**) in the culture supernatant were measured.

We first examined whether IL-4 stimulation induced Ccl17 and Ccl22 production in BMDCs. IL-4 stimulation upregulated Ccl17 and Ccl22 expression at the mRNA and protein levels in a dose-dependent manner (Figure 1a,b,e,f) and in a time-dependent manner (Figure 1c,d,g,h). These findings are consistent with those in a previous report [23].

### 2.2. Glyteer Treatment Inhibited IL-4-Induced Ccl17 and Ccl22 Production in BMDCs

**Figure 2:**
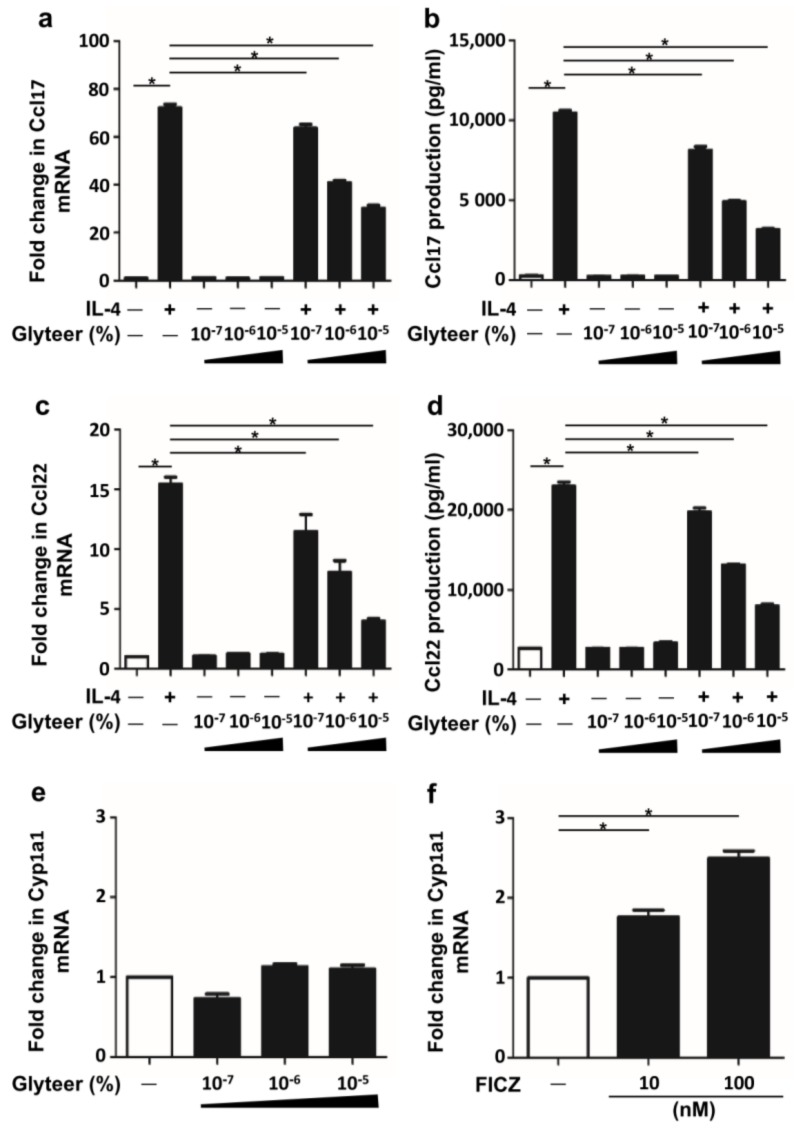
Glyteer treatment inhibited IL-4-induced Ccl17 and Ccl22 production in BMDCs. (**a**–**f**) Data are expressed as mean ± S.E.M.; *n* = 3 for each group; * *p* < 0.05. IL-4-simulated BMDCs were treated with Glyteer or 6-formylindolo[3.2-b]carbazole (FICZ) at the indicated dose for 24 h. Expression of *Ccl17* (**a**) and *Ccl22* (**c**) in BMDCs was analyzed by Quantitative Reverse Transcription (qRT)-PCR. Production of Ccl17 (**b**) and Ccl22 (**d**) in culture supernatant was measured by ELISA. Expression of *Cyp1a1* in BMDCs treated with Glyteer (**e**) or FICZ (**f**) was analyzed by qRT-PCR.

We examined the cytotoxicity of graded concentrations of Glyteer (up to 10^−4^%) for BMDCs. Because BMDCs were viable with Glyteer 10^−5^% (Figure S1), these concentrations were utilized in the experiments. Next, we examined whether Glyteer treatment affected IL-4-induced Ccl17 and Ccl22 production in BMDCs. For this, we pretreated BMDCs with Glyteer for 24 h and then stimulated them with IL-4 (10 ng/mL) for 24 h in the presence of Glyteer. Glyteer treatment inhibited IL-4-induced upregulation of Ccl17 and Ccl22 at the mRNA and protein levels in a dose-dependent manner (Figure 2a–d).

Because Glyteer treatment activates Ahr in keratinocytes [9,10], we also examined whether it activates Ahr in BMDCs. We analyzed the expression of Cyp1a1, a representative gene mediated by Ahr activation in DCs [24]. However, Glyteer treatment did not potently change the expression of Cyp1a1 (Figure 2e), while FICZ, an endogenous ligand of Ahr, significantly upregulated the expression of Cyp1a1 in BMDCs (Figure 2f). These results indicate that Glyteer treatment did not affect the Ahr activation in BMDCs, implying that the inhibitory effect of Glyteer treatment on IL-4-induced upregulation of Ccl17 and Ccl22 expression is likely to be Ahr-independent. 

### 2.3. Glyteer Treatment Inhibited IL-4-Induced Ccl17 and Ccl22 Production in an Ahr-Independent Manner in BMDCs

**Figure 3:**
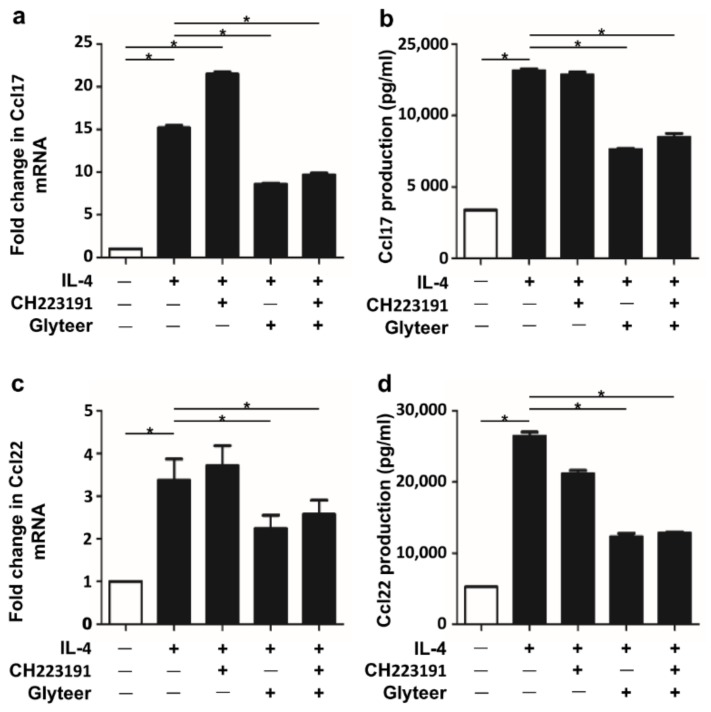
Glyteer treatment inhibited IL-4-induced Ccl17 and Ccl22 production in an aryl hydrocarbon receptor (Ahr)-independent manner in BMDCs. (**a**–**d**) Data are expressed as mean ± S.E.M.; *n* = 3 for each group; * *p* < 0.05. IL-4-stimulated BMDCs were treated with Glyteer in the absence or presence of CH223191 for 24 h. Glyteer and CH223191 were administered at the same timing. Expression of Ccl17 (**a**) and Ccl22 (**c**) in BMDCs was analyzed by qRT-PCR. Production of Ccl17 (**b**) and Ccl22 (**d**) in culture supernatant was measured by ELISA.

To further confirm the Ahr independence of the inhibitory action of Glyteer on IL-4-induced Ccl17 and Ccl22 production, we utilized CH223191, a selective Ahr antagonist. As shown in Figure 3a,b, the inhibitory action of Glyteer on the IL-4-induced Ccl17 upregulation was not canceled by CH223191 (10 μM) at both mRNA and protein levels. Similar results were obtained in IL-4-induced Ccl22 upregulation (Figure 3c,d), supporting the notion of an Ahr-independent inhibitory action of Glyteer.

### 2.4. Glyteer Treatment Inhibited Nuclear Translocation of Stat6 Induced by IL-4 Stimulation without Affecting Phosphorylation of Stat6 in BMDCs

**Figure 4:**
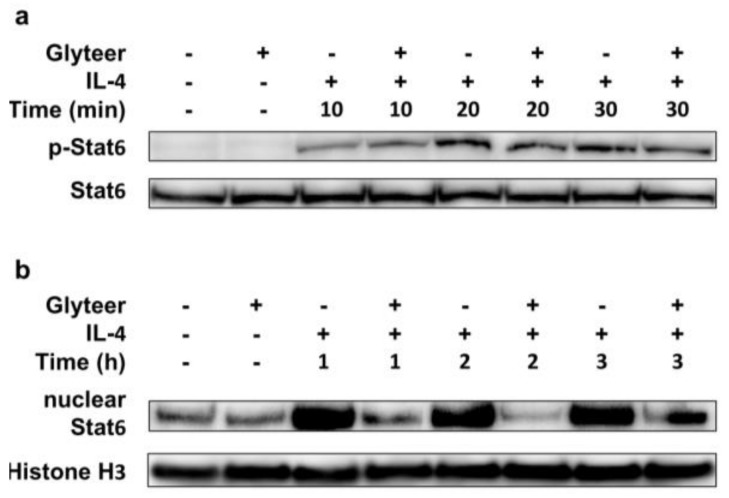
Glyteer treatment inhibited nuclear translocation of Stat6 induced by IL-4 stimulation without affecting the phosphorylation of Stat6 in BMDCs. (**a**) BMDCs treated with Glyteer for 24 h were stimulated with IL-4 (10 ng/mL) for 10, 20 and 30 min and then total protein or nuclear protein of the BMDCs was extracted; (**b**) BMDCs treated with Glyteer for 24 h were stimulated with IL-4 (10 ng/mL) for 1, 2 and 3 h and then total protein or nuclear protein of the BMDCs was extracted. Expression of phosphorylated Stat6 (pStat6) and Stat6 was analyzed by western blotting using anti-pStat6 antibodies. Histone H3 served as an internal loading control. The data are representative of experiments repeated three times with similar results.

To clarify the mechanism behind the inhibitory effect of Glyteer on the IL-4-induced Ccl17 and Ccl22 production, we examined whether Glyteer modulates the IL-4/Stat6 signal axis in BMDCs. The binding of IL-4 to IL-4 receptor (IL-4R) results in rapid tyrosine phosphorylation of STAT6 [25]. Phosphorylated STAT6 dimerizes and translocates to the nucleus where it acts as a transcription factor to regulate immune response-related genes such as Ccl17 and Ccl22 [26,27]. Here, BMDCs were pretreated with Glyteer 10^−5^% for 24 h and then stimulated with IL-4 (10 ng/mL) for the indicated time in the presence of Glyteer 10^−5^%. While Glyteer did not affect the phosphorylation or expression of Stat6 (Figure 4a), the nuclear translocation of Stat6 were reduced (Figure 4b), indicating that Glyteer treatment inhibited the nuclear translocation of phosphorylated Stat6 induced by IL-4 stimulation.

## 3. Discussion

Glyteer has been clinically used in Japan for 90 years as an alternative to coal tar because it is less viscous and less malodorous [9]. Although its beneficial effects on inflammatory skin diseases are known empirically, the fundamental mechanisms behind its effects have remained unclear throughout its time on the market [9]. Several studies including our own have shown that Ahr ligands such as Glyteer, FICZ and coal tar suppress IL-4-mediated inflammation and skin barrier dysfunctions in keratinocytes [8,9,10]. However, how Ahr ligands modulate the STAT6 signal pathway induced by IL-4 in dendritic cells has remained incompletely understood. There are two published reports stating that FICZ inhibits the phosphorylation of Stat6 in naïve CD4^+^ T cells [28] and that coal tar inhibits the oxidative inactivation of PTPN1, a negative regulator of STAT6, and thereby attenuates the phosphorylation of Stat6 in keratinocytes [8]; however, whether Ahr activation is involved in this has not been determined.

The present study has shown that Glyteer treatment resulted in reduced nuclear translocation of Stat6 induced by IL-4 stimulation, despite it affecting neither the phosphorylation nor the expression of Stat6. This indicates that Glyteer treatment inhibits the nuclear translocation of Stat6 but not its phosphorylation, leading to the inhibition of Ccl17 and Ccl22 production in BMDCs. Furthermore, this mechanism is likely to be independent of Ahr because Glyteer treatment did not affect the expression of *Cyp1a1*. Our recent study showed that FICZ interferes with TGF-β signaling in human fibroblasts in an Ahr-independent manner [29]. We have also reported that FICZ accelerates wound healing via ERK signaling but not via Ahr signaling in human keratinocytes [30]. These findings are consistent with another report describing that an endogenous aryl hydrocarbon Ahr ligand, 2-(1′*H*-indole-3′-carbonyl)-thiazole-4-carboxylic acid methyl ester (ITE), impaired TGF-β signaling in an Ahr-independent manner [31], indicating that Ahr ligands including Glyteer, FICZ and ITE have the ability to activate not only the canonical Ahr signaling pathway but also the non-canonical Ahr signaling pathway. Therefore, there is a possibility that Glyteer treatment activates non-canonical Ahr signaling, contributing to the impairment of IL-4/Stat6 signaling in BMDCs. However, further studies are required to confirm this.

Glyteer has been shown to be effective in the treatment of both psoriasis and AD [9,14]. Because Glyteer treatment inhibits IL-4/Stat6 signaling, in addition to its anti-inflammatory property, the utilization of Glyteer is thought to be more beneficial, especially in the treatment of AD. Furthermore, there is a possibility that such treatment potentiates the efficiency of dupilumab, an IL-4 receptor α-antagonist that inhibits IL-4/STAT6 signaling, in the treatment of AD. Dupilumab has recently been approved for use in the United States, Europe and Japan for the treatment of AD patients [32]. Dupilumab treatment has been shown to significantly improve clinical outcomes. It has also been reported that concomitant use of topical corticosteroids along with dupilumab results in greater improvement in the disease activity of AD than the use of dupilumab alone [32]. Therefore, combination therapy using Glyteer, topical corticosteroid and dupilumab may be beneficial for the treatment of AD.

## 4. Materials and Methods

### 4.1. Reagents

FICZ was purchased from Enzo Life Sciences (Exeter, UK). Glyteer was provided as an original stock solution by Fujinaga Pharm Co. Ltd. (Tokyo, Japan). Since Glyteer is dry distillation tar of delipidated soybean, it is thought to consist of a wide range of organic compounds and polycyclic aromatic hydrocarbons (PAHs), which is consistent with the fact that Glyteer activates the AHR signaling pathway [9,10]. However, no further attempts have been made to quantify the compound fraction of Glyteer. DMSO and CH-223191 were purchased from Sigma-Aldrich (St. Louis, MO, USA). Recombinant murine IL-4 was purchased from PeproTech (Rocky Hill, NJ, USA).

### 4.2. Generation of Bone Marrow-Derived DCs (BMDCs) and Cell Culture

C57BL/6 mice were housed in a clean facility, and bred and used in accordance with the guidelines of the animal facility center of Kyushu University. Bone marrow (BM) cells freshly isolated from the mice were cultured in RPMI 1640 medium (Sigma-Aldrich) supplemented with 10% FCS (Japan Bio Serum, Fukuyama, Japan), 10 mmol/L, 4-(2-hydroxyethyl)-1-piperazineethanesulfonic acid(HEPES) (10 mL; Invitrogen, Waltham, MA, USA), 1% Minimum Essential Medium Non-Essential Amino Acids (MEM NEAA) (10 mL; Invitrogen), 1 mmol/L sodium pyruvate (10 mL; Invitrogen), β-mercaptoethanol (50 nmol/L; Invitrogen), antibiotic-antimycotic 100× (5 mL; 100 U/mL penicillin, 100 mg/mL streptomycin and 0.25 µg/mL amphotericin B; Invitrogen) in the presence of GM-CSF (10 ng/mL) (Miltenyi Biotec, Bergisch Gladbach, Germany). On day 3, half of the culture medium was refreshed and GM-CSF was added. On day 7, non-adherent cells were harvested. These cells were purified immunomagnetically by two or three rounds of positive selection with CD11c (N418) MicroBeads (Miltenyi Biotec). As we described previously [33], the purity of DCs, as determined by fluorescence-activated cell sorting (FACS), was between 95% and 97%. Purified BMDCs were cultured with Glyteer and FICZ. Culture supernatants were collected at 24 h and analyzed by ELISA. Cells were also collected for PCR analysis or western blot analysis as described below.

### 4.3. WST-1 Assay

A WST-I assay was used to assess cell viability, in accordance with the manufacturer’s protocol (Clontech, Mountain View, CA, USA). BMDCs were treated with Glyteer at the indicated dose for 24 h. Optical density was measured using a DTX 800 Multimode Detector (Beckman Coulter, Brea, CA, USA).

### 4.4. Quantitative Reverse Transcription (qRT)-PCR Analysis

Total RNA was extracted using the RNeasy^®^ Mini kit (Qiagen, Venlo, The Netherlands). Reverse transcription was performed using PrimeScript^™^ RT reagent kit (Takara Bio, Otsu, Japan). qRT-PCR was conducted on a CFX Connect^™^ Real-time System (Bio-Rad, Hercules, CA, USA) using SYBR^®^ Premix Ex Taq (Takara Bio). Amplification was initiated at 95 °C for 30 s as the first step, followed by 40 cycles of qRT-PCR at 95 °C for 5 s and at 61 °C for 20 s. mRNA expression was measured in triplicate and mRNA levels normalized to β-actin were expressed as fold induction relative to the control group. The sequences of primers are shown in Table 1.

### 4.5. Western Blotting Analysis

Cells were incubated for 5 min in lysis buffer (Roche Diagnostics, Basel, Switzerland). The protein concentration in the lysate was measured using a BCA Protein Assay Kit (Thermo Fisher Scientific, Rockford, IL, USA). Equal amounts of protein (20 μg) were dissolved in NuPAGE LDS sample buffer (Invitrogen) and a 10% sample reducing agent (Invitrogen). The lysates were boiled at 70 °C for 10 min and then loaded into and subjected to electrophoresis in NuPAGE 4%–12% Bis-Tris gels (Invitrogen) at 200 V for 60 min. The proteins were then transferred onto polyvinylidene difluoride membranes (Invitrogen), which were blocked with WesternBreeze Blocker/Diluent (Invitrogen). The membranes were then probed with anti-phosphorylated Stat6 rabbit polyclonal antibody (tyrosine 641) (Abcam, Cambridge, UK), anti-Stat6 rabbit polyclonal antibody (Abcam) and anti-histone H3 rabbit polyclonal antibody (Cell Signaling Technology, Danvers, MA, USA) overnight at 4 °C. Horseradish peroxidase-conjugated anti-mouse IgG antibodies (Cell Signaling Technology) served as secondary antibodies. The visualization of protein bands was accomplished with the SuperSignal West Pico Chemiluminescent Substrate (Thermo Scientific) using the ChemiDoc touch imaging system (Bio-Rad).

### 4.6. Cellular Nuclear Protein Preparation for Western Blot Analysis

BMDCs were pretreated with FICZ or Glyteer for 24 h. Cellular nuclear protein was collected using NE-PER Nuclear and Cytoplasmic Extraction Reagents^®^ (Thermo Fisher Scientific). The nuclear Stat6 expression in BMDCs was analyzed by western blotting. Histone H3 served as an internal loading control.

### 4.7. ELISA

Murine Ccl17 and Ccl22 ELISA Kit (R&D Systems, Minneapolis, MN, USA) was used for ELISA, in accordance with the manufacturers’ protocol. Optical density was measured using a DTX 800 Multimode Detector (Beckman Coulter).

### 4.8. Statistical Analysis

Unpaired Student’s *t* test or one-way analysis of variance was used to assess the results. A *p*-value of <0.05 was considered to indicate a statistically significant difference. All data are presented as mean ± standard error of the mean (S.E.M.) from three independent experiments.

## Figures and Tables

**Table 1:** The sequences of primers.

Gene	Sequence (5’ to 3’)	
*β-actin*	forward	GGCTGTATTCCCCTCCATCG
	reverse	CCAGTTGGTAACAATGCCATGT
*Ccl17*	forward	AGGTCACTTCAGATGCTGCTC
	reverse	ACTCTCGGCCTACATTGGTG
*Ccl22*	forward	GACACCTGACGAGGACACA
	reverse	GCAGAGGGTGACGGATGTAG
*Cyp1a1*	forward	TCCTCCGTTACCTGCCTAACTC
	reverse	GATGTGGCCCTTCTCAAATGTCC

## Supplementary Materials

The following are available online at http://www.mdpi.com/1422-0067/19/4/1169/s1.

## Abbreviations

Ccl17	Chemokine (C-C motif) ligand 17
Ccl22	Chemokine (C-C motif) ligand 22
IL-4	Interleukin-4
Stat6	Signal transducer and activator of transcription 6
