# Supplementary material for: Glyteer, Soybean Tar, Impairs IL-4/Stat6 Signaling in Murine Bone Marrow-Derived Dendritic Cells: The Basis of Its Therapeutic Effect on Atopic Dermatitis
Source: Int J Mol Sci. 2018 Apr 12;19(4):1169. doi: 10.3390/ijms19041169 (PMC5979322; doi:10.3390/ijms19041169)
Supplement: Supplementary file 1 [file ijms-19-01169-s001.pdf]

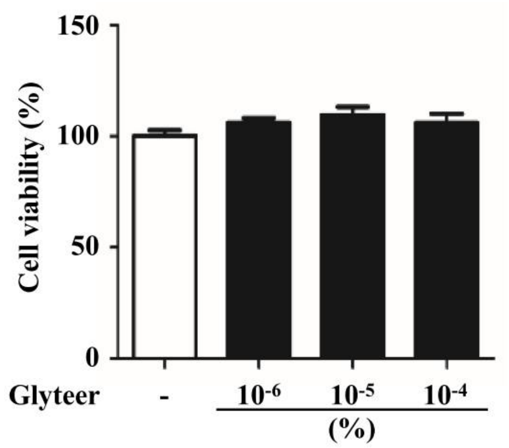

Figure S1 Cell viability of Glyteer-treated BMDCs. Data are expressed as mean ± S.E.M.; $n = 3$ for each group; * $p < 0.05$. BMDCs were treated with Glyteer at the indicated concentration. Cell viability was evaluated by WST-1 assay. The relative cell viability (%) compared with DMSO (control)-treated BMDCs was calculated.
